# Preimplantation Genetic Screening with Spent Culture Medium/Blastocoel Fluid for in Vitro Fertilization

**DOI:** 10.1038/s41598-018-27367-4

**Published:** 2018-06-18

**Authors:** Penghao Li, Zhe Song, Yaxin Yao, Tianhua Huang, Rurong Mao, Jun Huang, Yongyi Ma, Xin Dong, Wenlong Huang, Jihua Huang, Tianjian Chen, Ting Qu, Lingxiao Li, Ying Zhong, Jiang Gu

**Affiliations:** 1 Jinxin Research Institute for Reproductive Medicine and Genetics, Chengdu Jinjiang Hospital for Maternal and Child Health Care, 66 Jingxiu Road, Chengdu, 610066 China; 2 Laboratory of Molecular Pathology, Center of Molecular Diagnosis and Personalized Medicine, Provincial Key Laboratory of Infectious Diseases and Molecular Pathology, Shantou University Medical College, Shantou, China; 3 Department of Clinical Research, Yikon Genomics Co. Ltd., Building 26, 1698 Wangyuan Road, Fengxian District, Shanghai, 201499 China; 4 Department of Pathology, Beijing University Health Science Center, Beijing, China; 5 Hayward Genetics Center, Department of Pediatrics, Tulane University School of Medicine, New Orleans, LA USA

## Abstract

Preimplantation genetic screening (PGS) detects chromosomal aneuploidy from DNA extracted from trophectodermal biopsy of the embryos before implantation. Although a controlled study showed no difference in pregnancy rates between this invasive cell biopsy technique and a non-biopsied control group, the potential long-term damage by the current PGS method has not be completely ruled out. We therefore tested a less-invasive protocol which utilizes spent culture medium combining with blastocoel fluid (ECB) to assess chromosomal aneuploidy. We compared the new protocol with the currently employed trophectodermal biopsy method against chromosomal information obtained from the remaining embryo. We found that the new technique generated information about aneuploidy that was not entirely identical to obtained from the biopsied trophectoderm or the remaining embryo. As the origins of the DNA extracted from the three sample types were not the same, the significance and interpretation of each result would have its own meaning. The possible implications derived from the ECB results as well as those from cell biopsy were discussed. The effectiveness of this new approach in selecting the best embryo for uterine implantation awaits further long term evaluation.

## Introduction

Preimplantation genetic diagnosis (PGD) and preimplantation genetic screening (PGS) techniques for *in vitro* fertilization (IVF) were first developed by Handyside *et al*. in 1990^1^ and IVF has since evolved to include PGS/PGD leading to improved success rate particularly for genetically vulnerable and older populations^2–5^. In this technique, biopsy of one cell from day 3 or a few cells from day 5 or 6 embryo were physically performed under a dissecting microscope, and the genetic information of the collected cells was analyzed with fluorescence *in situ* hybridization (FISH), array comparative genomic hybridization (aCGH), single-nucleotide polymorphism (SNP) arrays, multiplex quantitative PCR or next generation sequencing (NGS) to determine the chromosomal status of the embryo to facilitate selection of desired embryos for implantation^6–10^. Such techniques, however, rely on the biopsy of a single cell or a few cells from the preimplantation embryo that is invasive to the developing embryo and its potential long-term harm to the off-spring has not been fully established^11^. It was reported that children born following PGS were found to have mild abnormalities with regard to fine motor function, posture and muscle tone at 18 months of age^12^. Two year old PGS children were shown to have subtle neurological deficiencies compared with controls^13^. A recent assessment of IVF deliveries in a multicenter obstetric and neonatal follow-up suggested that the risk of adverse obstetric and neonatal outcomes related to PGS/PGD was mainly related to the underlying parental condition rather than the PGS/PGD procedure^14^.

Although the controlled study by Scott *et al*.^15^ showed no difference in pregnancy rates in comparison with a non-biopsied control group in humans, animal studies showed that embryo biopsy influenced epigenetic reprogramming during early embryo development thus impacting neural development and function in resulting mice^16^. There were also reports suggesting that embryo biopsy influenced adrenal development and response to cold stress in mice^17^. In addition, the current procedure of trophectoderm cell biopsy is technically challenging, which has prevented this technique from widespread application^18^. Therefore, a less-invasive technique using spent culture medium and/or blastocoel fluid (ECB) of the embryo to assess the genetic and chromosomal defects is desirable. Efforts have been made to develop non-invasive method for PGS. The presence of DNA in the blastocoel fluid was first detected by Palini *et al*. in 2013^19^, and a pilot study using blastocentesis for preimplantation genetic testing was carried out in 2014^20^. Furthermore, Stigliani *et al*. demonstrated the presence of genomic and mitochondrial DNA in the embryo culture medium and their ratio can be a predictor of blastocyst potential and implantation outcome^21^. In addition, embryo culture medium-based noninvasive preimplantation genetic diagnosis for human alpha-Thalassemia was reported^22^. Recently, an attempt of non-invasive PGS was reported^23^ in which the authors performed whole genome PCR amplification of DNA extracted from spent embryo culture medium and then examined with NGS. They reported that the non-invasive technique obtained a high correlation to that obtained from the biopsy for detection of chromosomal aneuploidy (sensitivity: 88% and specificity: 84% respectively) but no comparison was made against the whole embryo or the remaining embryo.

Here, we report the development of a less-invasive technique using spent embryo culture medium/blastocoel fluid (ECB) to assess aneuploidy in the embryo and performed a comparative investigation of chromosomal analyses using ECB, biopsied cells and the remaining embryos. Following DNA extraction we employed a newly developed single cell DNA amplification protocol and NGS to evaluate the pros and cons of this new and less-invasive technique for possible embryo selection.

## Results

### DNA concentrations in the ECB were sufficiently high for DNA amplification, NGS and aneuploidy analysis

Forty (40) cultured embryos were investigated in our study. For ethical reasons, the embryos chosen were those that were not selected for implantation but had good morphological scores. Such embryos may have larger variations in their chromosomal status facilitating comparison of the three samples in our investigation. The criteria of embryo selection was based on recommendation by Gardner and Schoolcraft in 1999^24,25^. In each case, three protocols were performed, i.e. collecting ECB fluid, embryo cell biopsy and the remaining whole embryos. After whole genome amplification DNA extracted with each protocol yielded results of chromosomal information that is sufficient to analyze aneuploidy in the embryo.

The entire DNA obtained from the three samples was amplified separately with random primers in an amplification kit (Ref. No. YK001B, Yikon Genomics, China). Concentrations of the amplified products from different procedures were measured and analyzed by electrophoresis (Supplement Fig. S1). After amplification, the average concentrations of DNA obtained from three different sources were similar to one another, at 56.20 ng/ul for remaining embryo, 62.60 ng/ul for ECB and 58.03 ng/ul for biopsied cells, respectively (Table S1); all were above the level for subsequent sequencing. With blastocoel fluid alone, and less so with spent culture medium, we were unable to consistently generate sufficient amount of DNA for amplification and sequencing (Fig. S2). We found that when the concentration of DNA after amplification was below 10 ng/ml, NGS could not be performed.

### Specificity of the new test

**Figure 1:**
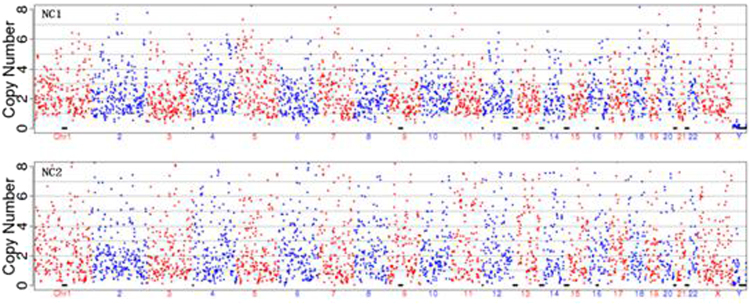
Negative controls with culture medium only gave completely negative result with splattering dots demonstrating that no DNA was successfully amplified.NC1: Culture medium processed identically to that of the spent medium but without embryo; NC2: Fresh culture medium.

The negative controls with unused culture medium gave completely negative result (Fig. 1). Among the 40 cases, 22 are female showing two X chromosomes, 17 male showing a single X and a single Y chromosome and one case showing one X no Y (X0). The three samples from each case gave identical results on sexual chromosomes. The entire procedure took less than 12 hours to complete including specimen sampling, DNA amplification, sequencing and analysis if time for sample transportation and handling was not counted.

### The new less-invasive method generated information of chromosomal aneuploidy for embryo analysis

**Figure 2:**
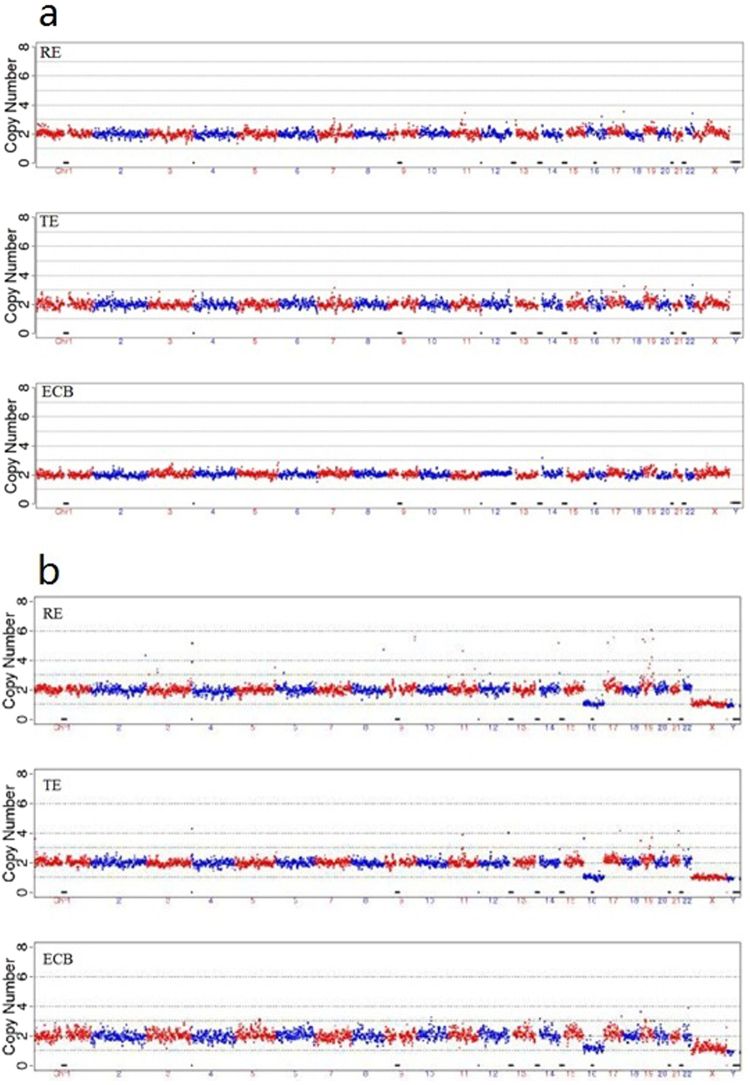
Examples of agreement among the three types of samples. (**a**) Example of results of all three sources showing identical chromosomal pattern with no aneuploidy. (**b**) Example of results of all three DNA sources, all showing aneuploidy for chromosomal 16.

**Figure 3:**
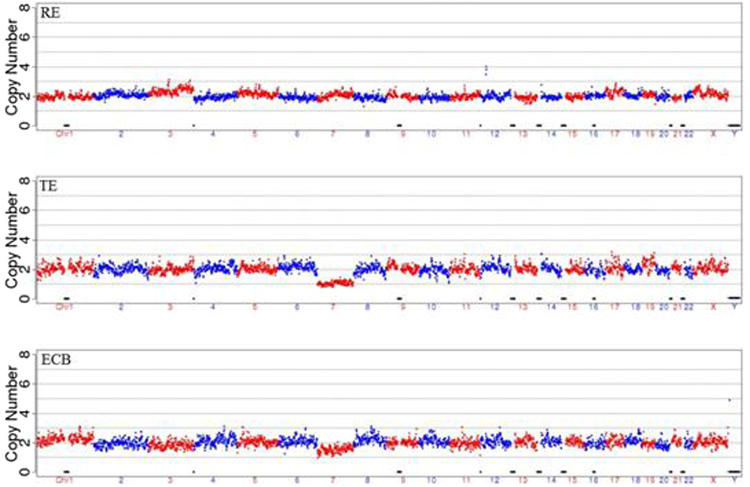
Results of the ECB method and the biopsy method were the same but were different from that of the remaining embryo. In this case, cell biopsy (TE) showed a clear chromosomal 7 aneuploidy but the remaining embryo (RE) had normal chromosomes while the embryo culture medium/blastocoel fluid (ECB) showed a half decrease at chromosome 7. Clearly, the result of cell biopsy did not represent that of the remaining embryo. It is likely that the aneuploidy only existed in the biopsied cells but not in the entire embryo. In this case, the culture medium/blastocoel fluid would be a better representation of the chromosomal dynamics of the entire embryo.

**Figure 4:**
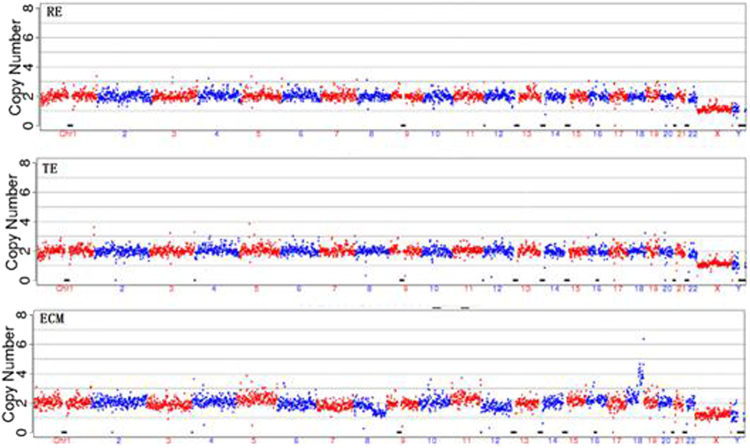
The biopsy method and the remaining embryo gave the same results but were different from that of the non-invasive (ECM) method. In this case, cell biopsy and remaining embryo demonstrated normal chromosomes but culture medium/blastocoel fluid showed additional abnormalities for chromosomes 8 and 18 suggesting that perhaps there were repairing activities at chromosomes 8 and 18 during development. Therefore this embryo would be less than ideal for implantation despite of the fact that cell biopsy gave a normal appearance. However, if there is a shortage of qualified embryos for this patient, this embryo can also be a candidate for consideration of implantation.

**Figure 5:**
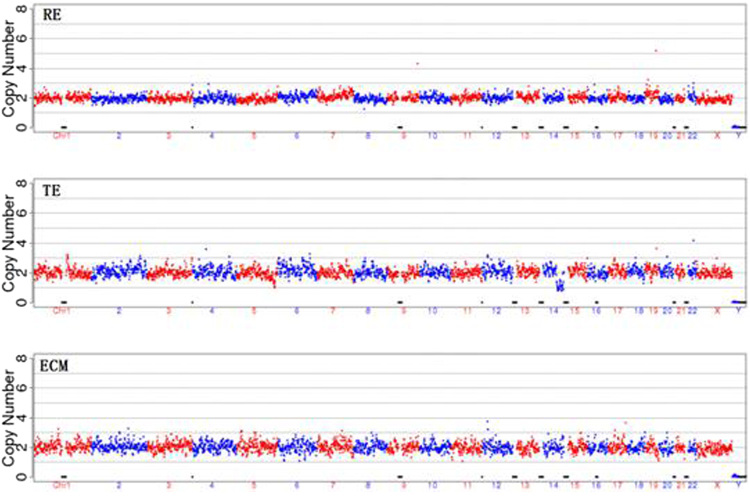
The non-invasive method (ECM) and the remaining embryo (RE) gave the same results but the biopsy method (TE) showed abnormality. In this case, the culture medium/blastocoel fluid and the remaining embryo gave the same results (both normal) but the cell biopsy method gave a clear aneuploidy for chromosome 14 showing that the biopsied cells may not always represent the entire embryo.

**Figure 6:**
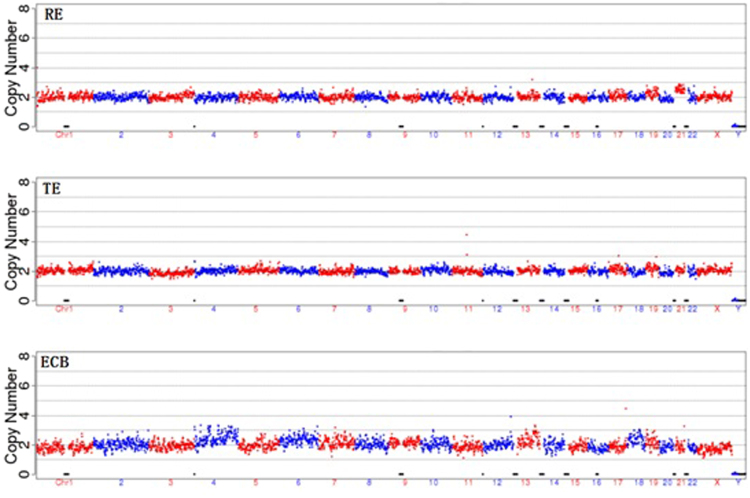
Three types of samples were in disagreement with one another. In this case, the remaining embryo showed a clear chromosomal 21 aneuploidy but the biopsy had normal chromosomes while the culture medium/blastocoel fluid showed a different aneuploidy chromosomal pattern.

The original results of our tests were presented in Figs 1–6 and Supplement Tables S2–S4. Three samples were not successfully amplified due to DNA disintegration for reasons unknown and were excluded from further analysis (one remaining embryo (ID: 9913-6), one cell biopsy samples (ID: 12943-4) and one ECB samples (ID: 14515-1)). Comparison of results obtained with the three types of samples gave five different sets of results (Table S2). In Group 1, which included 15 cases, the chromosomal patterns derived from all three types of samples were in agreement. Among these, 11 cases were normal (Fig. 2a) and 4 showed aneuploidy in all three samples (Fig. 2b). Group 2 had 2 cases in which the ECB method and the biopsy method were in agreement but differed from that of the remaining embryo (Fig. 3). Group 3 had 12 cases in which the biopsy method and the remaining embryo gave the same results but differed from that of the non-invasive method (Fig. 4). Group 4 had 4 cases in which the non-invasive method and the remaining embryo gave the same results but not the biopsy method (Fig. 5). Group 5 included 7 cases in which three types of samples were in disagreement with one another (Fig. 6).

Using the chromosome status of the remaining whole embryos as the standard, we calculated the sensitivity and specificity of the cell biopsy protocol and the less-invasive (ECB) method (Tables S3-S4). In total there are 38 cases for the comparison between biopsy and the remaining embryo and the comparison between ECB and the remaining embryo. The sensitivity was calculated as follows: No. of True Positives/(No. of True Positives + No. of False Negatives); The specificity was calculated as follows: No. of True Negatives/(No. of True Negatives + No. of False Positives); Therefore the biopsy method had a sensitivity of 89.47% (17/(17 + 2)) and specificity of 73.68% (14/(14 + 5)) while the ECB method had a sensitivity of 89.47% (17/(17 + 2)) and specificity of 68.42% (13/(13 + 6)) (Tables S5-S6).

The data were also analyzed at the individual chromosome level. In comparison to the remaining embryo as the standard, the biopsy method had a sensitivity of 83.33% (25/(25 + 5)) and specificity of 97.63% (824/(824 + 20)), while the ECB method had a sensitivity of 59.38% (19/(19 + 13)) and specificity of 95.84% (807/(807 + 35)) (Tables S5-S6).

### The commonly employed cell biopsy method and the newly developed less-invasive ECB method did not always give the same results to that of the remaining embryo

It should be noted that in 11 cases (27.5%), the cell biopsy method generated different results from that of the remaining embryo although both samples were taken from the same embryos at the same time. In 20 cases (50%), the ECB method obtained different results from that of the remaining embryo. In 22 cases (55%), the biopsy and the ECB methods gave different results (Table S2).

## Discussion

Our investigation showed that the mixture of spent culture medium and blastocoel fluid contains sufficient amount of DNA that can be amplified and sequenced by NGS (Fig. S1). It demonstrated that ECB of 5 day embryo could generate sufficient DNA for aneuploidy detection. We lasered the zona pellucida and released the blastocoel fluid into the culture medium to increase the concentration of embryo DNA^26^. This procedure systemically released the blastocoel fluid without causing any harm to the embryo. In fact, Mukaida *et al*. reported that artificial shrinkage of blastocoels by micro-needle or a laser pulse before vitrification improved the survival rate and clinical outcome of the embryo^27^. The reliability of the single cell DNA amplification and sequencing technique employed in this study has been well established in evaluating chromosomal aneuploidy in previous reports^23,28,29^_._

We noted that the results obtained with ECB were not exactly the same to that obtained with cell biopsy or with the remaining embryo. This can be explained by a number of possibilities. The three sample types represent different status of the chromosomes in the embryo (Table S2). DNA in the biopsied cells only yielded information of the removed cells at the particular time of removal that sometimes may not represent the remaining embryo, as has been shown in 11 cases of this study. The biopsied cells would not reflect any chromosomal errors occurred during period before the biopsy was taken or occurred in other cells. The whole remaining embryo reflects the DNA status of the entire embryo at the particular time of sampling, but not changes in the embryo occurred before day 5. Chromosome information from the remaining embryo would reflect alternations of many cells and proivde an average of them. On the other hand, the ECB contained DNA released by embryo cells into the spent medium and during blastocoel formation from day 3 to day 5 as the culture medium was changed at day 3 and the blastocyst was only formed from day 3 onwards. As was shown in this study, the ECB gave a somewhat different chromosomal patterns to that of the biopsied cells as it might reflect changes of the entire embryo from days 3–5. This difference might also be a reflection of mosaicism which was reported in embryos of animals and humans^3,30^. Many studies have been conducted on this topic and the reported mosaicism rates varied from 13% to 38% in humans^31–37^. In a review by Jannie van Echten-Arends, mosaicism was reported to occur in 73% of the embryos with various forms. The most common pattern was euploid-aneuploid mosaicism where the embryo contained a complement of both normal and abnormal cells. The same report indicated that more mosaicism occurred at the blastula stage than at the cleavage stage. The higher percentage of diploid cells in diploid–aneuploidy blastocysts compared with cleavage-stage embryos (74% versus 62%) might indicate that normal cells would tend to survive and abnormal cells tend to be eliminated^38^. The disagreement in results obtained among ECB, cell biopsy and the remaining embryo could be a reflection of the rates of mosaicism of the embryos at this stage. In addition, the fact that, for ethical reasons, the embryos used in this study were not the best embryos, which were used for implantation or frozen storage, might also explain the relatively high discordance among the results of the three protocols.

It should be noted that previous studies reported much higher sensitivity and specificity when comparing biopsy and the remaining embryo^10,35,39–44^. We do not have a ready explanation for this discrepancy but many factors might have contributed to these differences. Previous studies used different techniques and they mostly analyzed the total gains and losses of entire chromosomes while we employed NGS which had a much higher resolution than FISH or aCGH. In some study comparisons were made between different techniques, i.e. aCGH vs. FISH, or aCGH vs. NGS^35,41^. In a similar study, the whole blastocysts was analyzed to compare against the initial biopsy using the same aCGH protocol. However it examined only whole chromosome gains and losses, and in addition, the embryos they selected should have better morphological scores than ours. Therefore, we speculate that the real differences in their study should be higher than what was reported^44^. Chromosome data generated from the whole embryo would reflect all abnormalities of all the cells and the average of them. The resolution of NGS would unveil many more variations than those by FISH or aCGH. Although the rate of mosaicism in our samples (11/40) is similar to those reported by others^31–37^, we discarded any mosaicism less than 30% and above 80%, therefore the actual mosaicism rate could be higher. The only comparable study was reported by Xu *et al*. in which the sensitivity and specificity were 88% and 84% respectively, which are comparable to ours^23^. However, in their study, comparison was made only between cell biopsy and spent culture medium, but the remaining embryos were not examined nor compared. Of course, the different technical protocols used in different studies could be responsible for the differences in our results and those of the previous reports. It remains to be seen what future studies of similar nature will unveil in terms of sensitivity and specificity of this technique.

Our study also revealed the limitation and bias produced by both cell biopsy and the ECB procedures. In 4 cases, the chromosomal pattern derived from cell biopsy was different from those of the remaining embryo and the ECB while the latter two were in agreement to each other. In 12 cases, ECB generated different results from the remaining embryos and the cell biopsy while the latter two were in agreement to each other. In another 7 cases, none of the three methods generated results agree to one another. This result showed that the cell biopsy and the new ECB procedures, although mostly accurate, could mislead in selecting the right embryo for implantation.

We also attempted to amplify DNA from the blastocoel fluid or the spent culture medium. Although previous studies reported that human blastocoel fluid or spent culture medium contained PCR-amplifiable DNA^19,20^, in our study we found that DNA extracted from blastocoels fluid was not enough for amplification and sequencing (Fig. S2). DNA extracted from the spent culture fluid could generate enough DNA for amplification but the amounts were not consistent. The difference between our results and that by Xu *et al*. could come from slightly different protocols^23^. We felt that pooling of the blastocoel fluid and the spent culture medium would be a better technique for aneuploidy detection.

Possible contamination of the spent culture fluid by protein supplement of the culture medium, which has a major binding affinity for DNA, is a concern^26^. This should not be a problem in our protocol as fresh culture medium was used as a negative control which showed that baseline DNA contamination did not interfere with the final results. Mitochondrial DNA was filtered out in our amplification and sequencing. Contamination by cumulus cells was also very unlikely which would gave a skewed female dominance in sex distribution but in our study the two sexes were largely balanced. In addition, in our experiments the sex chromosomes were always in agreement among the three sample sources in each case further suggesting no contamination from maternal cumulus cells as biopsied embryo cells were very unlikely to contain such cells. We were careful in washing away any cumulus cells during ova collection and fertilization. In addition, we changed culture medium at day 3 instead of using a continuous culture medium protocol as used in a previous report that raised the concern of cumulus cell contamination^26^. In addition, we sequenced the amplified DNA at 0.02~0.03 depth and only detected DNA sequence above 50 MB in size that does not provide sufficient resolution for detailed analysis of genetic abnormalities but only for chromosomal aneuploidy. More accurate genetic analysis could be achieved by increasing the sequencing depth and with targeted sequencing.

The less-invasive technique we developed has a number of potential advantages over the currently employed cell biopsy method. Most importantly, it causes less harm to the embryo and there would be little concern of potential damage to the developing embryo and the IVF baby. Release of the blastocoel fluid would not amount to any damage to the embryo and has been routinely used in the frozen embryo IVF technique. The obvious bias and limitation of the biopsied cells as well as the new EBC method in representing the remaining embryo are demonstrated in our experiment. As chromosomal mosaicism is a common phenomenon in developing embryo^31–37^, it is possible that the biopsied cells contained defective chromosomes but cells on average of the remaining embryo do not. The opposite may also be true. In addition, the protocol of collecting ECB is much easier to perform than cell biopsy which is a challenging technique requiring highly trained and experienced individuals to perform. By only collecting ECB, human errors in cell biopsy can be avoided. Possible contamination of the sample is also reduced to the minimum due to much less manipulation of the embryo in the procedure. The protocol can be completed within one day. The new protocol would make the new technique more applicable by IVF laboratories worldwide.

Our experiment demonstrated that ECB analysis by DNA amplification and NGS can generates aneuploidy data for IVF. However, the differences in results between the ECB and the TE methods and between both and the remaining embryo poses a challenge for explanation. Interpretation of the ECB method would be more complicated as the DNA of this protocol may come from apoptotic cells and shedding from developing embryo cells for various reasons from days 3–5. The low sensitivity and specificity of our protocol indicates that this technique is not yet ready for clinical application. The usefulness of this new method awaits for further optimization and long term evaluation.

## Materials and Methods

A total of 40 preimplantation embryos at day 5 were examined. The embryos were donated by couples seeking for IVF at Jinjiang Women and Children Hospital, Chengdu, China. Ethical approval was obtained from the Scientific and Ethical Committee of Jinjiang Women and Children Hospital for the experimental protocol (2016KY-005). The embryos chosen for this study were those that were left-over from implant and frozen but also had good morphological scores. The procedure of ova collection, *in vitro* fertilization, embryo culture, and implantation followed the standard hospital protocol (CDXN/QD-EMBYO-02-17) that has been approved by the Ethical Committee of Jinjiang Hospital which was accredited by ISO9001 and JCI (Joint Commission on Accreditation of Healthcare Organizations). Patient consent was obtained for each embryo sample used in this study.

### Gamete preparation and ICSI procedure

#### Oocyte preparation

The patients were injected with HCG 36 hours before obtaining ova via vagina under ultrasound. The ova were washed and then placed into 0.3 ml of culture medium G-MOPS Plus (Ref. No. 10130, Vitrolife, Sweden) and 0.3 ml 80 u/ml hyaluronidase (Ref. No. ART-4007-A, SAGE, US) medium with repeated pipetting within 30 seconds and then transferred to culture medium G-MOPS (Ref. No. 10129, Vitrolife, Sweden) without HSA. The procedure was performed according to the manufacture’s instruction. To completely remove granulosa cells, the cultured ova was continuously agitated using a pipette with the help of the stickiness of the culture medium G-MOPS PLUS with HSA. When the granulosa cells were completely removed, the ova was transferred into new culture medium G-IVF PLUS (Ref. No. 10136, Vitrolife, Sweden) covered with OVOIL (Ref. No. 10029 Vitrolife, Sweden). The culture dish was incubated in 37 °C in an incubator containing 6% CO_2_ for 1–1.5 hours.

#### Sperm preparation

Sperms were placed under inverted microscope at 200 or 400 times magnification. Sperm morphology was assessed according to the protocol recommended by World Health Organization (WHO) criteria (2010). Normal sperms have a smooth, oval configuration with a well-defined acrosome incorporating 40–70% of the sperm head, and with no neck, mid-piece or tail defects and no cytoplasmic droplets more than one-half the size of the sperm head. Sperms of good morphology were selected and immobilized with the injection pipette.

#### ICSI procedure

The preparation of the holding and injection pipettes has been described in detail elsewhere^45–47^. Only metaphase II oocytes were injected. Oocytes were held by the holding pipette with polar body at the 12 o’clock position and the injection pipette was inserted into the oocyte at the 3 o’clock position^48^.

### Embryo culture

Following ICSI, each ova was placed in a micro-culture well Embryoscope Culture Dish (Vitrolife, Sweden) containing 25 µl G-1 PLUS (REF. No. 10029, Vitrolife, Sweden) culture medium and then into Embryoscope Time-Lapse Incubator (Vitrolife, Sweden). At day 3 the culture medium was removed and changed into G-2 PLUS (REF. No. 10132, Vitrolife, Sweden) and it was cultured to day 5 until the formation of blastocyst^49^.

### Sample collection

#### Embryo culture medium and blastocoel fluid

An infrared laser (Hamilton Thorne Biosciences, Beverley, MA) was used to lase a small breech in the zone pellucida (ZP) to release the blastocoels fluid into the culture medium. The location of the breach was far away from the inner cell mass. After the embryo was removed, the released blastocoel fluid mixed with culture media (≈25 µl) was transferred to RNase–DNase-free PCR tubes and named Group 1. To prevent media contamination, different Pasteur pipettes were used for each sample.

#### Trophectoderm cells (TE)

The blastocysts were then placed individually in a biopsy dish containing 20 µL of G-MOPS PLUS (REF. No. 10130, Vitrolife, Sweden) under oil for biopsy. Trophectoderm cells were encouraged to herniate from the zona by applying gentle suction with the biopsy pipette. Three to five trophectoderm cells were dissected from each of the blastocysts using four laser pulses of 3 seconds duration^46,47^. The biopsied cells were placed immediately in RNase–DNase-free PCR tubes, and named Group 2 (TE).

#### Remaining embryo (RE)

Following the above biopsy the remaining embryo was placed into RNase–DNase-free PCR tubes and named Group 3 (RE).

#### Negative control (NC)

The culture medium identically processed but did not contain embryo was used as a negative control (NC1) and unused culture medium was also used as a negative control (NC2).

### Whole genome amplification (WGA)

The biopsied cells, the culture medium and the remaining embryo were all subjected to MALBAC single-cell WGA following the manufacture’s protocol (catalog no. YK001B; YIKON Genomics) to amplify the DNA^28,29^. Briefly, the cell was lysed by heating (90 min at 50 °C and 10 min at 80 °C) in 5 μL of lysis buffer. Then 30 μL of freshly prepared pre-amplification mix was added to each tube and was incubated at 94 °C for 3 min. Then DNA was amplified using eight cycles of 40 s at 20 °C, 40 s at 30 °C, 30 s at 40 °C, 30 s at 50 °C, 30 s at 60 °C, 4 min at 70 °C, 20 s at 95 °C, and 10 s at 58 °C and was placed on ice immediately. We then added 30 μL of the amplification reaction mix to each tube and incubated at 94 °C for 30 s followed by 17 cycles of 20 s at 94 °C, 30 s at 58 °C, and 3 min at 72 °C. Took 5 μL of the amplification product to run electrophoresis (1% agarose gel, 110 V, 25–35 min). The amplification product is 300–2000 bp. The amplification product was then purified and quantified with Nanodrop. The final product is 2–5 μg.

### NGS and analysis

The sequencing libraries were constructed with the amplified DNA using NEB Next Ultra DNA Kit (New England Biolabs, UK)and were then sequenced with a Illumina HiSeq 2500 platform, yielding about 2 million sequencing reads on each sample. The sequencing depth is about 0.02~0.03x and was carried according to a procedure described previously^39,50^.

The read numbers were counted along the whole genome with a bin size of 1.5~2 Mb. A copy number gain from 2 to 3 copies results in a 50% increase in read counts, while a copy number loss from 2 copies to 1 copy results in a 50% decrease in read counts.

### Detailed data analysis method

Raw sequencing reads from the sequencing library were trimmed with Trimmomatic-0.30^51^ adapters and low quality bases (quality score less than 20) were removed. High quality reads were aligned to the University of California, Santa Cruz, human reference genome (hg19) (http://genome.ucsc.edu/) using the Burrows–Wheeler Aligner 0.7.4^52^ with default parameters. The aligned reads were sorted with Picard 1.92 (http://picard.sourceforge.net/). The chromosomal copy number variations were determined with local Perl (http://www.perl.org/) scripts; unique mapped reads were normalized to relative reads number after GC correction in 1000 Kb bins. The visualization of copy number variations was performed with R programming language (http://www.r-project.org/).

## Electronic supplementary material

Supplementary Information
