# Supplementary material for: Preimplantation Genetic Screening with Spent Culture Medium/Blastocoel Fluid for in Vitro Fertilization
Source: Sci Rep. 2018 Jun 18;8:9275. doi: 10.1038/s41598-018-27367-4 (PMC6006313; doi:10.1038/s41598-018-27367-4)
Supplement: Supplementary file 1 — Supplementary Information [file 41598_2018_27367_MOESM1_ESM.docx]

**Preimplantation Genetic Screening with Spent Culture Medium/Blastocoel Fluid for in Vitro Fertilization**

Penghao Li^1#^, Zhe Song^2#^, Yaxin Yao^3#^, Tianhua Huang^1^, Rurong Mao^1^, Jun Huang^1^, Yongyi Ma^1^, Xin Dong^1^, Wenlong Huang^2^, Jihua Huang^1^, Tianjian Chen^5^, Ting Qu^1^, Lingxiao Li^1^, Ying Zhong^1^, Jiang Gu^1, 2, 4^

| Supplement Table S1. The average concentrations of DNA after amplification obtained from three different sources | | | |
| --- | --- | --- | --- |
|  | RE ng/μl | TE ng/μl | ECB ng/μl |
| Mean±SD | 56.20±20.33 | 62.60±30.45 | 58.03±35.87 |
| Median | 58.79 | 59.99 | 55.34 |

Variables are shown as mean ± SD and median.

RE: Remaining embryo after cell biopsy; TE: Trophectoderm cell biopsy; ECB: Culture medium/blastocoel.

Supplement Figure S1. Electrophoresis of amplified DNA from the three sample types and negative controls.


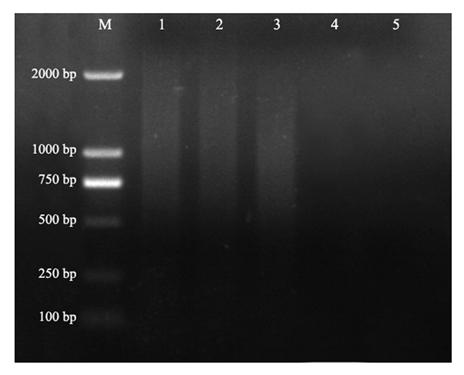


The size of DNA products ranges from 300~2000bp. All three sample sources generated enough DNA after amplification for sequencing.

Lane M: DNA marker; Lane 1: remaining embryo; Lane 2: biopsied cells; Lane 3: mixture of culture medium and blastocoel fluid; Lane 4: negative control (culture medium processed identically but without embryo within); Lane 5: negative control (fresh culture medium).

Supplement Figure S2. Electrophoresis of amplified DNA from the blastocoel fluid, culture medium, mixture of culture medium and blastocoel fluid.


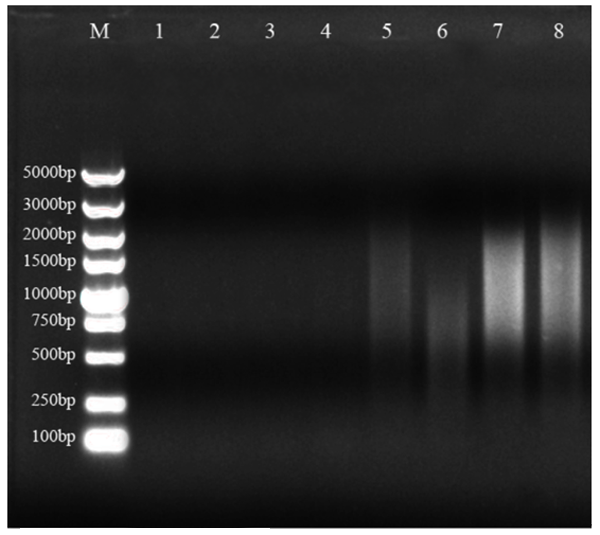


Lane M: DNA marker; Lane 1: negative control (culture medium processed identically but without embryo within); Lane 2: negative control (fresh culture medium); Lane 3 and Lane4：blastocoel fluid only; Lane 5 and Lane 6：culture medium only; Lane 7 and Lane 8：mixture of culture medium and blastocoel fluid

Supplement Table S2. Comparison of results obtained from the three sample types

| Three types of samples were in agreement (n=15) | | | |
| --- | --- | --- | --- |
| Sample ID | Remaining embryo | Cell biopsy | ECB |
| 11494-4 | 46,XX | 46,XX | 46,XX |
| 14468-5 | 46,XY | 46,XY | 46,XY |
| 14468-9 | 46,XY | 46,XY | 46,XY |
| 14282-2 | 46,XX | 46,XX | 46,XX |
| 19540-10 | 46,XY | 46,XY | 46,XY |
| 13082-3 | 46,XX | 46,XX | 46,XX |
| 13126-10 | 46,XX | 46,XX | 46,XX |
| 13744-6 | 46,XX | 46,XX | 46,XX |
| 13744-7 | 46,XX | 46,XX | 46,XX |
| 11586-1 | 46,XX | 46,XX | 46,XX |
| 14358-6 | 46,XX | 46,XX | 46,XX |
| 13326-2 | 45,XY,-8(×1) | 45,XY,-8(×1) | 46,XY,-8(×1,mos,~40%) |
| 11586-7 | 45,XY,-16(×1) | 45,XY,-16(×1) | 45,XY,-16(×1) |
| 13268-10 | 45,XY,+1p(pter→p35.3,~31M,×3),+4(pter→q23,~101M,×3),-7(×1) | 45,XY,+1p(pter→p35.3,~29M,×3,mos,~30%),+4(pter→q23,~101M,×3),-7(×1) | 45,XY,+1p(pter→p35.3,~31M,×3),+4(pter→q23,~101M,×3),-7(×1) |
| 13860-4 | 46,XY,+2(×3,mos,~30%),+7(×3,mos,~30%),+10(×3,mos,~30%) | 46,XY,+2(×3,mos,~30%),+7(×3,mos,~30%),+10(×3,mos,~30%) | 46,XY,+2(×3,mos,~30%),+7(×3,mos,~30%),+10(pter→q22.1,~73M,×3,mos,~30%) |
| Cell biopsy and ECB were in agreement (n=2) | | | |
| 9913-6 | - | 46,XX | 46,XX |
| 12825-6 | 46,XX,+3(q22.1→qter,~68M,×3,mos,~30%),+17q(q12→qter,~45M,×3,mos,~30%) | 45,XX,-7(×1) | 46,XX,-7(×1,mos,~50%) |
|  |  |  |  |
| Cell biopsy and remaining embryo were in agreement (n=12) | | | |
| 13268-8 | 47,XX,+1p(pter→p35.3,~28M,×3),-2(p12→qter,~160M,×1),-4q(q23→qter,~90M,×1),+16(×3) | 47,XX,+1p(pter→p35.3,~28M,×3),-2(p12→qter,~160M,×1),-4q(q23→qter,~90M,×1),+16(×3) | 47,XX,+9(×3) |
| 13268-9 | 46,XX,-1p(pter→p35.2,~32M,×1),+4q(q23→qter,~91M,×3),-11p(pter→p12,~43M,×1,mos,~30%) | 46,XX,-1p(pter→p35.2,~32M,×1),+4q(q23→qter,~91M,×3),-11p(pter→p12,~43M,×1,mos,~30%) | 46,XX, +4q(q13.2→qter,~121M, ×3),+5q(q15→q34,~75M,×3,mos,~30%),+17(pterq24.2,~67M,×3,mos,~30%) |
| 13326-3 | 45,XY,-4(×1) | 45,XY,-4(×1) | 47,XY,+1(×3),+11(×3,mos,~30%),+12(×3,mos,~30%),+13(×3,mos,~30%) |
| 13922_9 | 46,XY | 46,XY | 46,XY, +12(pter→q14.1, ~58M,×3),-21(×1,mos,~60%) |
| 13209-4 | 46,XX,+8q(q21.3→qter,~50M,×3) | 46,XX,+8q(q21.3→qter,~50M,×3) | 46,XX,-9(×1,mos,~50%) |
| 11494-7 | 45,XY,-22(×1) | 45,XY,-22(×1) | 46,XY |
| 14515-1 | 45,XY,-10(×1) | 45,XY,-10(×1) | - |
| 14515-2 | 46,XY | 46,XY | 46,XY,-8q(q21.13→qter,~60M,×1,mos,~30%),+18q(q21.2→qter,~26M,×4) |
| 19596-5 | 46,XY | 46,XY | Multiple chromosomal abnormalities |
| 13007-2 | 44,X,-X(×1),-15(×1) | 44,X,-X(×1),-15(×1) | 44,X,-X(×1),+14(×3,mos,~30%),-15(×1) |
| 14277-8 | 45,XX,-16(×1) | 45,XX,-16(×1) | 45,XX,-16(×1),+17(×3,mos,~30%) |
| 12871-8 | 46,XX,-16(×1,mos,~50%) | 45,XX,-16(×1) | 46,XX,-16(pter→q12.1,~51M,×1),+16q(q12.2→qter,~35M,×3,mos,~30%) |
| ECB and remaining embryo were in agreement (n=4) | | | |
| 12825-9 | 46,XX | 46,XX,-14q(q24.3→qter,~32M,×1) | 46,XX |
| 12871-4 | 46,XY | 46,XY,+2q(q23.3→q33.1,~53M,×3,mos,~30%),+15q(q22.2→q26.1,~31M,×3),+20(p11.21→qter,~37M,×3), +22(×3,mos,~30%) | 46,XY |
| 12943-4 | 46,XY, +9q(q21.11→q22.2，~23M,×3),+15(×3,mos,~30%),+19(×3,mos,~30%) | - | 46,XY, +9q(q21.11→q22.2，~23M,×3),+15(pter→q26.1,~71M,×3,mos,~50%) |
| 14437-4 | 46,XX,-18q(q12.2→qter,~43M,×1,mos,~40%) | Multiple chromosomal abnormalities | 46,XX,-18q(q21.1→qter,~35M,×1,mos,~50%) |
| Three types of samples were not in agreement (n=7) | | | |
| 13887_3 | 46,XX | 46,XX,-1(p21.13→qter,~143M,×1),+4(p15.33→q34.1,~161M,×3),-5(×1),+10(×3),-X(×1,mos,~30%) | 47,XX,+6(×3),+15(×3, mos,~30%),+17(×3, mos,~30%), +18(×3,mos,~30%),+22(×3, mos,~30%) |
| 12825-7 | 46,XX,+21(×3,mos,~50%) | 46,XX | 46,XX, +4(×3,mos,~50%) |
| 12825-8 | 46,XY | 46,XY,+1p(pter→p36.11,~20M,×3) | 46,XY,+1p(pter→p36.11,~27M,×5),+4q(q22.1→qter,~103M,×6),-9(×1,mos,~30%) |
| 12871-10 | 46,XX,+19(×3,mos,~50%) | 46,XX,+3q(q13.13→qter,~90M,×3,mos,~50%) | 45,XX,-2(×1,mos,~30%),-22(×1) |
| 14437-10 | 46,XY,-6(×1,mos,~40%) | 46,XY | 46,XY,+6p(pter→p12.1,~55M,×5) |
| 13268-12 | 46,XX | 46,XX,+19(×3,mos,~50%) | 46,XX,+10(×3,mos,~30%),+13(×3,mos,~30%) |
| 14304-3 | 45,XX,-10(×1) | 45,XX,-10(×1),-16q(q11.2→qter,~42M,×1) | 46,XX |

Note: “–”indicates that DNA sample was not amplifiable due to disintegration. Multiple chromosomal abnormalities: Chromosomal abnormalities were too numerous to be listed in the table. ECB；embryo culture medium/blastocoele fluid

Supplement Table S3. Summary of results obtained from cell biopsy versus corresponding remaining embryo

| Normal embryos (Cell biopsy and remaining embryo consistent)(n=14) | | |
| --- | --- | --- |
| Sample ID | Remaining embryo | Cell biopsy |
| 11494-4 | 46,XX | 46,XX |
| 14468-5 | 46,XY | 46,XY |
| 14468-9 | 46,XY | 46,XY |
| 14282-2 | 46,XX | 46,XX |
| 19540-10 | 46,XY | 46,XY |
| 13082-3 | 46,XX | 46,XX |
| 13126-10 | 46,XX | 46,XX |
| 13744-6 | 46,XX | 46,XX |
| 13744-7 | 46,XX | 46,XX |
| 11586-1 | 46,XX | 46,XX |
| 14358-6 | 46,XX | 46,XX |
| 13922_9 | 46,XY | 46,XY |
| 14515-2 | 46,XY | 46,XY |
| 19596-5 | 46,XY | 46,XY |
|  |  |  |
| False-positive embryos (Cell biopsy abnormal, remaining embryo normal)(n=5) | | |
| 13268-12 | 46,XX | 46,XX,+19(×3,mos,~50%) |
| 12825-9 | 46,XX | 46,XX,-14q(q24.3→qter,~32M,×1) |
| 12871-4 | 46,XY | 46,XY,+2q(q23.3→q33.1,~53M,×3,mos,~30%),+15q(q22.2→q26.1,~31M,×3),+20(p11.21→qter,~37M,×3), +22(×3,mos,~30%) |
| 13887_3 | 46,XX | 47,XX,-1(p21.13→qter,~143M,×1),+4(p15.33→q34.1,~161M,×3),-5(×1),+10(×3),-X(×1,mos,~30%) |
| 12825-8 | 46,XY | 46,XY,+1p(pter→p36.11,~20M,×3) |
|  |  |  |
| Abnormal embryos (Cell biopsy and remaining embryo consistent)(n=17) | | |
| 13007-2 | 44,X,-X(×1),-15(×1) | 44,X,-X(×1),-15(×1) |
| 13326-2 | 45,XY,-8(×1) | 45,XY,-8(×1) |
| 11586-7 | 45,XY,-16(×1) | 45,XY,-16(×1) |
| 13268-10 | 45,XY,+1p(pter→p35.3,~31M,×3),+4(pter→q23,~101M,×3),-7(×1) | 45,XY,+1p(pter→p35.3,~29M,×3,mos,~30%),+4(pter→q23,~101M,×3),-7(×1) |
| 12871-8 | 46,XX,-16(×1,mos,~50%) | 45,XX,-16(×1) |
| 13860-4 | 46,XY,+2(×3,mos,~30%),+7(×3,mos,~30%),+10(×3,mos,~30%) | 46,XY,+2(×3,mos,~30%),+7(×3,mos,~30%),+10(×3,mos,~30%) |
| 14277-8 | 45,XX,-16(×1) | 45,XX,-16(×1) |
| 12825-6 | 46,XX,+3(q22.1→qter,~68M,×3,mos,~30%)，+17q(q12→qter,~45M,×3,mos,~30%) | 45,XX,-7(×1) |
| 13268-8 | 47,XX,+1p(pter→p35.3,~28M,×3),-2(p12→qter,~160M,×1),-4q(q23→qter,~90M,×1),+16(×3) | 47,XX,+1p(pter→p35.3,~28M,×3),-2(p12→qter,~160M,×1),-4q(q23→qter,~90M,×1),+16(×3) |
| 13268-9 | 46,XX,- 1p(pter→p35.2,~32M,×1)+4q(q23→qter,~91M,×3),-11p(pter→p12,~43M,×1,mos,~30%) | 46,XX,-1p(pter→p35.2,~32M,×1),+4q(q23→qter,~91M,×3),-11p(pter→p12,~43M,×1,mos,~30%) |
| 13326-3 | 45,XY,-4(×1) | 45,XY,-4(×1) |
| 13209-4 | 46,XX,+8q(q21.3→qter,~50M,×3) | 46,XX,+8q(q21.3→qter,~50M,×3) |
| 11494-7 | 45,XY,-22(×1) | 45,XY,-22(×1) |
| 14515-1 | 45,XY,-10(×1) | 45,XY,-10(×1) |
| 14304-3 | 45,XX,-10(×1) | 45,XX,-10(×1),-16q(q11.2→qter,~42M,×1) |
| 12871-10 | 46,XX,+19(×3,mos,~50%) | 46,XX,+3q(q13.13→qter,~90M,×3,mos,~50%) |
| 14437-4 | 46,XX,-18q(q12.2→qter,~43M,×1,mos,~40%) | Multiple chromosomal abnormalities |
|  |  |  |
| False-negative embryos (Cell biopsy normal, remaining embryo abnormal)(n=2) | | |
| 12825-7 | 46,XX,+21(×3,mos,~50%) | 46,XX |
| 14437-10 | 46,XY,-6(×1,mos,~40%) | 46,XY |

Note: DNA from two samples were disintegrated and excluded from the calculation. Therefore the total is 38 cases.

Supplement Table S4 Summary of results obtained from ECB versus corresponding remaining embryo

| Normal embryos (ECB and remaining embryo consistent)(n=13) | | |
| --- | --- | --- |
| Sample ID | Remaining embryo | ECB |
| 11494-4 | 46,XX | 46,XX |
| 14468-5 | 46,XY | 46,XY |
| 14468-9 | 46,XY | 46,XY |
| 14282-2 | 46,XX | 46,XX |
| 19540-10 | 46,XY | 46,XY |
| 13082-3 | 46,XX | 46,XX |
| 13126-10 | 46,XX | 46,XX |
| 13744-6 | 46,XX | 46,XX |
| 13744-7 | 46,XX | 46,XX |
| 11586-1 | 46,XX | 46,XX |
| 14358-6 | 46,XX | 46,XX |
| 12825-9 | 46,XX | 46,XX |
| 12871-4 | 46,XY | 46,XY |
|  |  |  |
| False-positive embryos (ECB abnormal, remaining embryo normal)(n=6) | | |
| 13268-12 | 46,XX | 46,XX, +10(×3,mos,~30%),+13(×3,mos,~30%) |
| 13922_9 | 46,XY | 46,XY, +12(pter→q14.1,~58M,×3),-21(×1,mos,~60%) |
| 14515-2 | 46,XY | 46,XY,-8q(q21.13→qter,~60M,×1,mos,~30%),+18q(q21.2→qter,~26M,×4) |
| 13887_3 | 46,XX | 47,XX,+6(×3),+15(×3, mos,~30%),+17(×3, mos,~30%), +18(×3,mos,~30%),+22(×3, mos,~30%) |
| 12825-8 | 46,XY | 46,XY,+1p(pter→p36.11,~27M,×5),+4q(q22.1→qter,~103M,×6),-9(×1,mos,~30%) |
| 19596-5 | 46,XY | Multiple chromosomal abnormalities |
|  |  |  |
| Abnormal embryos (ECB and remaining embryo consistent)(n=17) | | |
| 13007-2 | 44,X,-X(×1),-15(×1) | 44,X,-X(×1),+14(×3,mos,~30%),-15(×1) |
| 13326-2 | 45,XY,-8(×1) | 46,XY,-8(×1,mos,~40%) |
| 11586-7 | 45,XY,-16(×1) | 45,XY,-16(×1) |
| 13268-10 | 45,XY,+1p(pter→p35.3,~31M,×3),+4(pter→q23,~101M,×3),-7(×1) | 45,XY,+1p(pter→p35.3,~31M,×3),+4(pter→q23,~101M,×3),-7(×1) |
| 12871-8 | 46,XX,-16(×1,mos,~50%) | 46,XX,-16(pter→q12.1,~51M,×1), +16q(q12.2→qter,~35M,×3,mos,~30%) |
| 13860-4 | 46,XY,+2(×3,mos,~30%),+7(×3,mos,~30%),+10(×3,mos,~30%) | 46,XY,+2(×3,mos,~30%),+7(×3,mos,~30%),+10(pter→q22.1,~73M,×3,mos,~30%) |
| 14277-8 | 45,XX,-16(×1) | 45,XX,-16(×1),+17(×3,mos,~30%) |
| 12825-6 | 46,XX,+3(q22.1→qter,~68M,×3,mos,~30%)，+17q(q12→qter,~45M,×3,mos,~30%) | 46,XX,-7(×1,mos,~50%) |
| 13268-8 | 47,XX,+1p(pter→p35.3,~28M,×3),-2(p12→qter,~160M,×1),-4q(q23→qter,~90M,×1),+16(×3) | 47,XX,+9(×3) |
| 13268-9 | 46,XX,-1p(pter→p35.2,~32M,×1),+4q(q23→qter,~91M,×3),-11p(pter→p12,~43M,×1,mos,~30%) | 46,XX, +4q(q13.2→qter,~121M,3),+5q(q15→q34,~75M,×3,mos,~30%),+17(pterq24.2,~67M,×3,mos,~30%) |
| 13326-3 | 45,XY,-4(×1) | 46,XY,+1(×3), +11(×3,mos,~30%), +12(×3,mos,~30%), +13(×3,mos,~30%) |
| 13209-4 | 46,XX,+8q(q21.3→qter,~50M,×3) | 46,XX,-9(×1,mos,~50%) |
| 12943-4 | 46,XY, +9q(q21.11→q22.2,~23M,×3),+15(×3,mos,~30%),+19(×3,mos,~30%) | 46,XY, +9q(q21.11→q22.2，~23M,×3),+15(pter→q26.1,~71M,×3,mos,~50%) |
| 14437-4 | 46,XX,-18q(q12.2→qter,~43M,×1,mos,~40%) | 46,XX,-18q(q21.1→qter,~35M,×1,mos,~50%) |
| 12825-7 | 46,XX,+21(×3,mos,~50%) | 46,XX, +4(×3,mos,~50%) |
| 12871-10 | 46,XX,+19(×3,mos,~50%) | 45,XX,-2(×1,mos,~30%),-22(×1) |
| 14437-10 | 46,XY,-6(×1,mos,~40%) | 46,XY,+6p(pter→p12.1,~55M,×5) |
|  |  |  |
| False-negative embryos (ECB normal, remaining embryo abnormal)(n=2) | | |
| 11494-7 | 45,XY,-22(×1) | 46,XY |
| 14304-3 | 45,XX,-10(×1) | 46,XX |

Note: DNA from two samples were disintegrated and excluded from the calculation. Therefore the total is 38 cases.

| Supplement Table S5 |  |  |  |
| --- | --- | --- | --- |
| Cell biopsy concordance analysis performance on remaining embryo | | |  |
| Samples | No. | (%) |  |
| Chromosome calling comparison^1^ | 874(23×38) |  |  |
| Euploid chromosomes (true negatives) | 824 |  |  |
| Aneuploid chromosomes (true positives) | 25 |  |  |
| Missed chromosomes (false negatives) | 5 |  |  |
| Extra chromosomes (false positives) | 20 |  |  |
| Performance |  |  |  |
| Sensitivity^2^ |  | 83.33% |  |
| Specificity^3^ |  | 97.63% |  |
|  |  |  |  |
| Whole-embryo aneuploidy/euploidy status comparison^1^ | 38 |  |  |
| Euploid embryo (true negatives) | 14 |  |  |
| Aneuploid embryo (true positives) | 17 |  |  |
| Missed aneuploid embryo (false negatives) | 2 |  |  |
| Extra aneuploid embryo (false positives) | 5 |  |  |
| Performance |  |  |  |
| Sensitivity^2^ |  | 89.47% |  |
| Specificity^3^ |  | 73.68% |  |
| Note: 1: DNA from one remaining embryo (ID: 9913-6) and one cell biopsy samples (ID: 12943-4) were disintegrated and excluded from the calculation. Therefore the total is 38 cases; 2.Sensitivity: No. of True Positives/(No. of True Positives + No. of False Negatives); 3.Specificity: No. of True Negatives/(No. of True Negatives + No. of False Positives); | | | |

| Supplement Table S6 |  | |  | | |
| --- | --- | --- | --- | --- | --- |
| ECB concordance analysis performance on remaining embryo | | | | |  |
| Concordance analysis | | No. | | (%) |  |
| Chromosome calling comparison^1^ | | 874(23×38) | |  |  |
| Euploid chromosomes (true negatives) | | 807 | |  |  |
| Aneuploid chromosomes (true positives) | | 19 | |  |  |
| Missed chromosomes (false negatives) | | 13 | |  |  |
| Extra chromosomes (false positives) | | 35 | |  |  |
| Performance | |  | |  |  |
| Sensitivity^2^ | |  | | 59.38% |  |
| Specificity^3^ | |  | | 95.84% |  |
|  | |  | |  |  |
| Whole-embryo aneuploidy/euploidy status comparison^1^ | | 38 | |  |  |
| Euploid embryo (true negatives) | | 13 | |  |  |
| Aneuploid embryo (true positives) | | 17 | |  |  |
| Missed aneuploid embryo (false negatives) | | 2 | |  |  |
| Extra aneuploid embryo (false positives) | | 6 | |  |  |
| Performance | |  | |  |  |
| Sensitivity^2^ | |  | | 89.47% |  |
| Specificity^3^ | |  | | 68.42% |  |
| Note: 1: DNA from one remaining embryo (ID: 9913-6) and one ECB samples (ID: 14515-1) were disintegrated and excluded from the calculation. Therefore the total is 38 cases; 2.Sensitivity: No. of True Positives/(No. of True Positives + No. of False Negatives); 3.Specificity: No. of True Negatives/(No. of True Negatives + No. of False Positives); | | | | |  |

| Table S7 The concentrations of DNA after amplification obtained from different samples | | | | | |
| --- | --- | --- | --- | --- | --- |
| Sample | Blastocoele fluid alone ng/μl | Media alone ng/μl | ECB ng/μl | NC ng/μl | PC ng/μl |
| 1 | 10.518 | 48.326 | 47.637 | 13.46 | 70.39 |
| 2 | 9.69 | 63.213 | 65.661 | 8.37 |  |
| 3 | 12.11 | 55.112 | 66.902 |  |  |
| Mean±SD | 10.77±1.23 | 55.55±7.45 | 60.07±10.78 |  |  |

Figure S3 Electrophoresis of amplified DNA from diferent samples and negative controls.


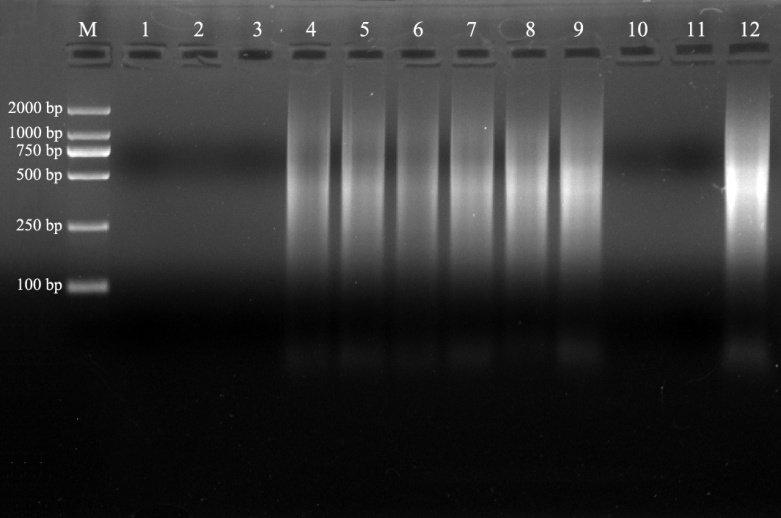


The size of DNA products ranges from 300~2000bp.

Lane M: DNA marker; Lane 1-3: blastocoele fluid alone; Lane 4-6: media alone; Lane 7-9: mixture of culture medium and blastocoel fluid(ECB); Lane 10: negative control (culture medium processed identically but without embryo within); Lane 11: negative control (fresh culture medium) ; Lane 12: positive control(cell biopsy).
